# A Case of Idiopathic First Bite Syndrome, Possibly Linked to Type I Diabetes Mellitus

**DOI:** 10.7759/cureus.36710

**Published:** 2023-03-26

**Authors:** Methodios T Stavridopoulos, Stavros N Nikitopoulos, Anastasia A Oikonomou, Georgia K Tsiouma

**Affiliations:** 1 Otolaryngology - Head and Neck Surgery, General Hospital of Volos, Volos, GRC; 2 Faculty of Medicine, University of Thessaly, Larisa, GRC

**Keywords:** diabetic autonomic neuropathy, type i diabetes mellitus, ipp, fbs, idiopathic parotid pain, first bite syndrome

## Abstract

We present a rare case of a 34-year-old male with poorly regulated type I diabetes and three-month history of excruciating pain in the right condylar process of the mandible, occurring only during the first bite of each meal. The patient had no history of surgery or trauma in the head and neck region. Clinical and imaging examination revealed no tumor or pathology deriving from the dentures, the temporomandibular joint (TMJ), or the salivary glands. Idiopathic first bite syndrome (FBS) was suspected and treated with pregabalin and glycemic control. This case highlights how a detailed pain history and clinical examination can lead to a rare diagnosis and indicates the potential involvement of diabetic neuropathy in idiopathic FBS, as well as the importance of glycemic regulation in treatment.

## Introduction

First bite syndrome (FBS) is defined as paroxysmal pain in the parotid area, precipitated by the first bite of each meal and declining as the meal progresses to complete resolution [1]. It is mainly a postoperative complication of head and neck surgery that is traditionally appointed to dissection of the sympathetic innervation of the parotid gland, or an unusual symptom of tumors arising in the same region [2]. Idiopathic FBS and idiopathic parotid pain (IPP) have been reported a few times and a link to type II diabetes has recently been suggested [3].

## Case presentation

A 34-year-old male presented to the ENT department with a three-month history of dysphagia due to severe pain in the right parotid area, radiating to the right periauricular region. The pain was characterized as paroxysmal, 10/10 on the Pain Numerical Rating Scale, occurred at the first bite of each meal immediately when the bolus reached the posterior section of the tongue, diminished in severity with subsequent bites and resided completely a few minutes after completion of the meal. The intensity of the pain was stronger with the consumption of acid foods but irrelevant to the time of day a meal was consumed. He had been treated three months prior, with anti-inflammatory agents, due to suspicion of TMJ disorder, with no benefit.

The patient suffered from poorly regulated type I diabetes mellitus and described occasional abdominal bloating and constipation that responded well to dietary changes. He reported no history of injury or surgery in the head and neck region. His vitals and ECG were normal, as was a complete blood count test except for high blood glucose and hemoglobin A1c levels (11.89%, normal range 4%-6.2%).

Throughout the clinical examination, no signs of the pathology of the salivary glands, masticatory muscles, teeth, temporomandibular joint (TMJ), or external auditory canal (EAC) were observed. Tenderness was evoked on mild palpation of the right parotid gland, not in the same intensity as the pain described, but not of the TMJ. Milking the parotid glands resulted in clear saliva secretion, with no pain. The pain was only provoked when a sour solution was applied to the right section of the tongue and not when biting a cotton swab or through jaw movements. The function of all cranial nerves was evaluated as normal and flexible fiberoptic nasopharyngolaryngoscopy revealed no pathology. Lastly, examination of the ipsilateral eye revealed a normal pupillary light reflex and no ptosis of the upper eyelid nor enophthalmos.

**Figure 1:**
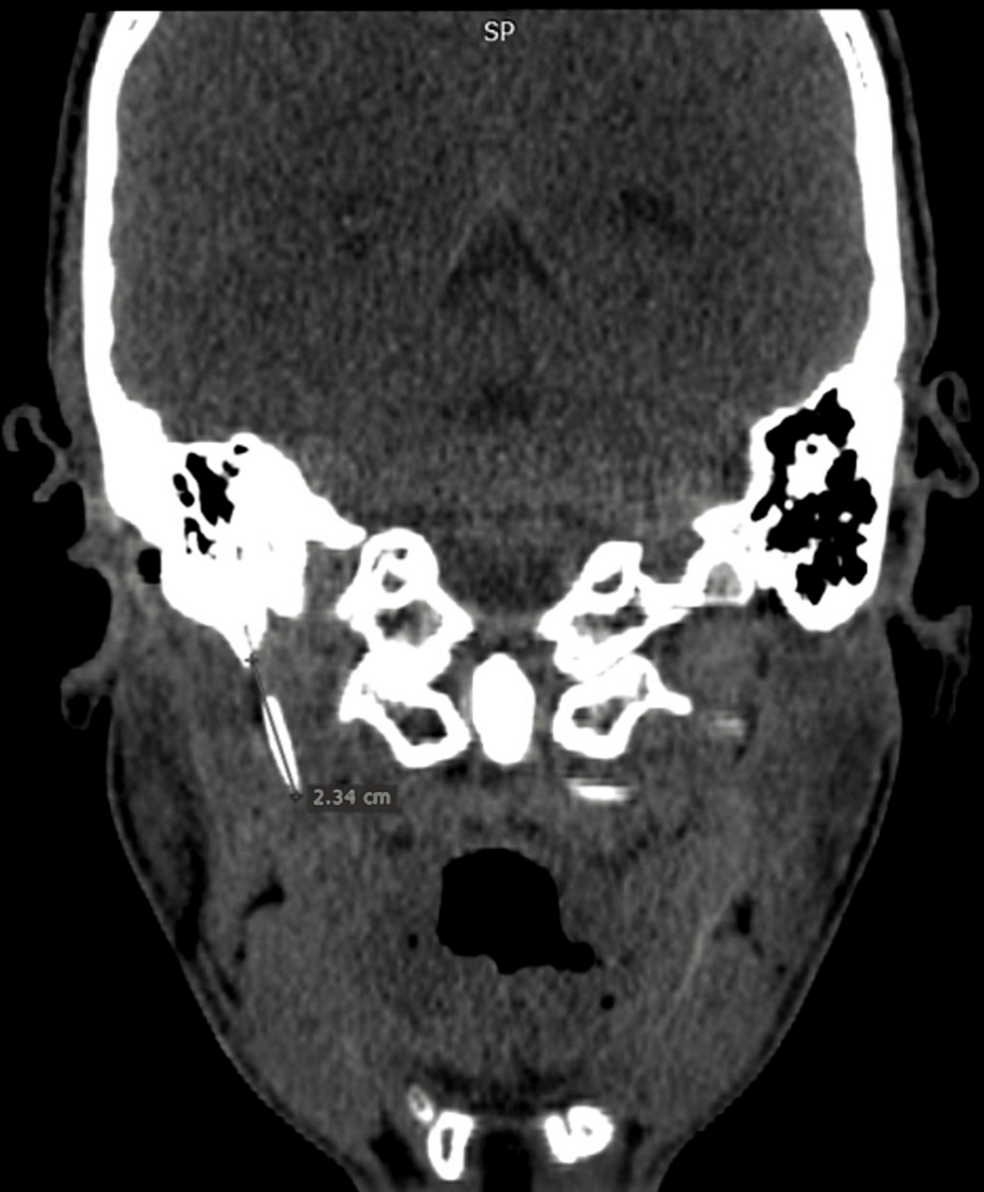
Coronal CT showing length of right styloid process within normal range

**Figure 2:**
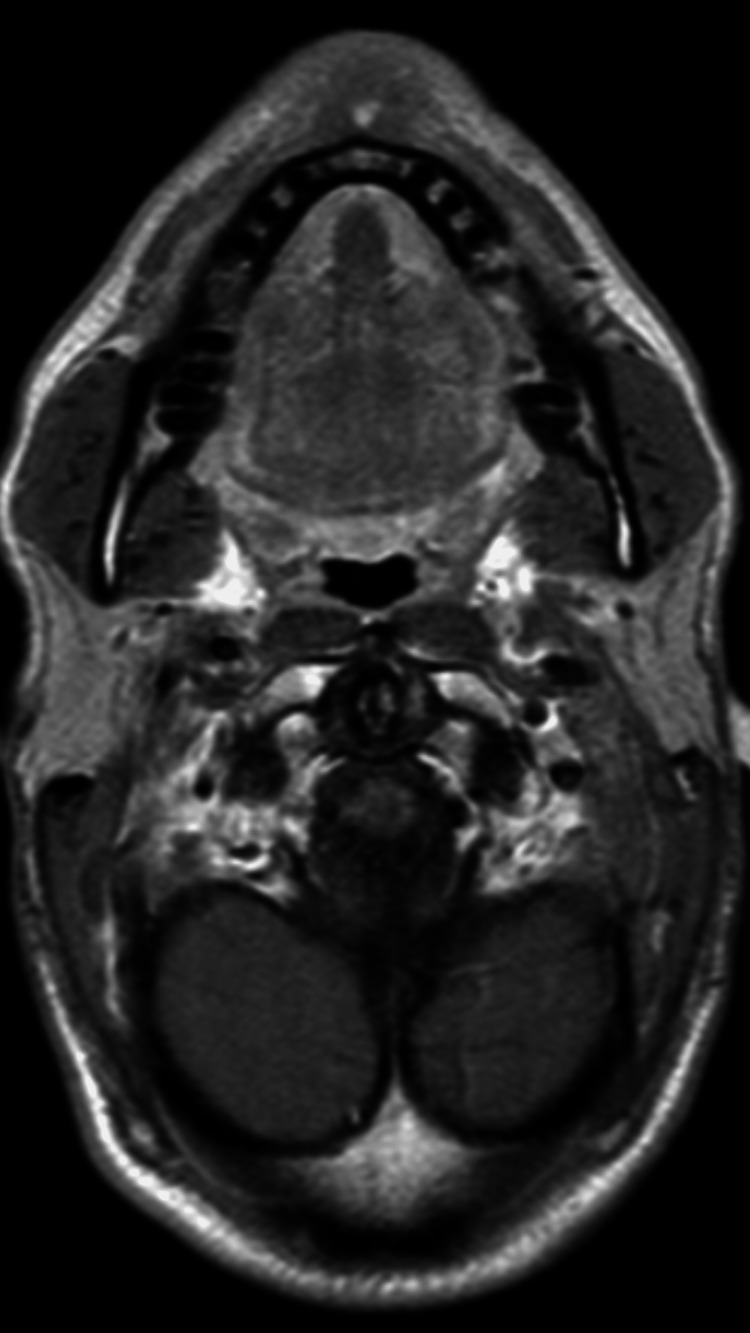
Axial MRI T1w showing no abnormalities arising from the left parotid gland or parapharyngeal space (PPS)

**Figure 3:**
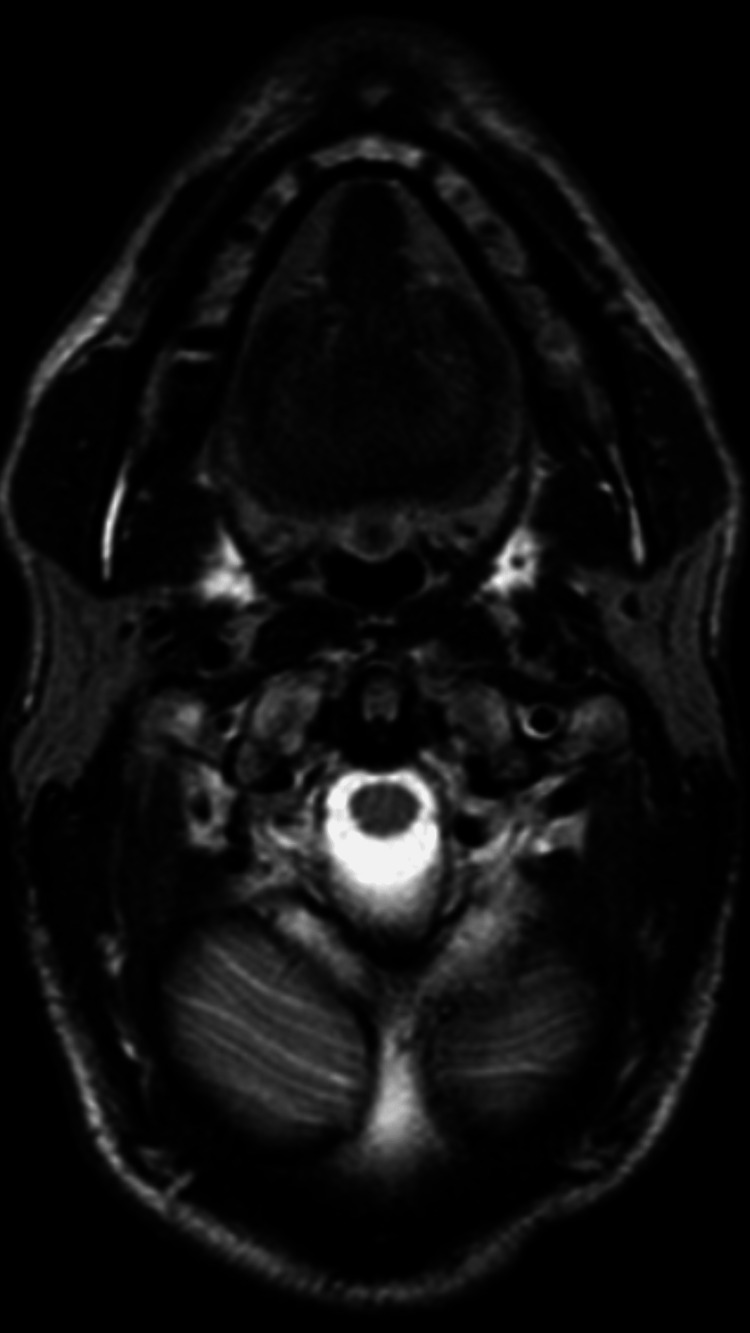
Axial MRI T2w showing no abnormalities arising from the left parotid gland or parapharyngeal space (PPS)

The patient underwent a panoramic x-ray, computed tomography (CT), and magnetic resonance imaging (MRI) of the head and neck, in order to exclude Eagle syndrome and tumors arising from the salivary glands or parapharyngeal space (PPS), as can be seen in Figures 1-3.

No findings or abnormalities were evident to support any of the above and thus idiopathic FBS was suspected. The patient was prescribed pregabalin 75mg twice per day at first, which after one week was increased to 150mg twice per day due to persistence of the pain, and was strongly advised to regulate diabetes. Follow-up took place two months later; the patient’s symptoms had resolved and his blood sugar levels were normal. Dose tapering instructions were provided and phone-call follow-up after one month ensured the patient remained asymptomatic. The patient skipped the radiological follow-up after six months willingly.

## Discussion

The term “first bite syndrome” was first used by W.S. Haubrich in 1986 to describe clinical features characterized by the occasional onset of pharyngeal blockage of food at the first bite of a meal, without prodromal symptoms and sometimes accompanied by retrosternal chest pain [4]. In 1998, Netterville et al. again used this term to characterize an early postoperative pain syndrome occurring in patients who underwent surgery for vagal paraganglioma [1]. The pain was severe, located in the parotid area, had sudden onset during the first bite of each meal, and subsided over the next bites. It appeared in the early postoperative period in the ipsilateral side of the removed vagal paraganglioma [1]. Gardner and Abdullah were the first who, in 1955, reported facial pain with characteristics much like the ones described as FBS, regarding patients with superior cervical ganglionectomy [5]. Authors have since embraced Netterville’s description.

The prevailing theory for the pathogenesis of FBS is the derangement of the autonomous innervation of the parotid gland’s myoepithelial cells [1]. Postoperative FBS has been reported in head and neck surgeries involving the PPS, the infratemporal fossa (ITF), the deep lobe of the parotid gland, the carotid space, the retropharyngeal space, the masticator space, and even after TMJ replacement [2]. Rarely, tumors originating from the aforementioned spaces can manifest FBS even if they are not detectable during an initial radiological examination. The pain in these cases is attributed to the denervation of the parotid’s sympathetic innervation, due to compression or infiltration, and is resolved with surgical removal of the mass. A follow-up scan should take place after six to nine months, as FBS may precede radiological detection of the mass [2].

Idiopathic FBS has rarely been reported in the literature. In most cases, these patients have been misdiagnosed at first. The similarity in pain pattern is what connects idiopathic FBS to traditional FBS. Differential diagnosis typically includes TMJ dysfunction, gastro-esophageal reflux, Eagle syndrome, and trigeminal or glossopharyngeal neuralgia [2]. TMJ dysfunction does not constitute a diagnostic problem, as jaw movements and palpation of the TMJ do not cause pain in these patients. Gastro-esophageal reflux is mainly characterized by digestive symptoms such as retrosternal chest pain and regurgitation. Eagle syndrome can be excluded by radiological findings and pain caused through palpation of the styloid process. Trigeminal neuralgia typically requires cutaneous trigger points, such as teeth brushing or combing of hair, located in areas innervated by branches of the trigeminal nerve. Glossopharyngeal neuralgia manifests as a stabbing pain in the tonsillar region of the oral cavity, not the parotid area.

Recently, the term IPP has been recommended [3]. Three differences between IPP and FBS have been highlighted 1) the possibility of bilateral IPP, 2) the sensitivity of the parotid glands to mechanical stimuli, and 3) the postprandial pain. IPP has been correlated with type II diabetes, in a manner of diabetic autonomic neuropathy (DAN) contributing to IPP in male patients with diabetes [3]. Diabetic cardiac autonomic neuropathy (CAN) is the most studied of the DANs and its prevalence increases with diabetes duration, up to 30% after 20 years [6]. Hyperglycemia is thought to be the primary factor [7].

In our case, although the criteria of IPP were not fully met, we believe that diabetic neuropathy was involved, as the improvement of blood glucose levels was relevant to pain management. Furthermore, our patient experienced occasional abdominal bloating and constipation which are considered gastrointestinal manifestations of DAN. Signs and symptoms from the cardiovascular and urogenital systems compatible with DAN were not present in our case.

It is commonly accepted that NSAIDs and dietary modifications are not an efficient treatment for FBS. Spontaneous resolution has been observed in post-operative FBS, but the patient always seeks medical help in the meantime. Surgical treatments such as dissection of the auriculotemporal nerve or tympanic neurectomy have had mixed results. Anticonvulsants (gabapentin, carbamazepine, and pregabalin) prescribed with or without amitriptyline are most often used but do not always completely alleviate the pain, or relapses are reported during dosage de-escalation. External adjuvant radiotherapy has been reported to treat postoperative FBS in oncologic patients. Lastly, intraparotid injections of botulinum toxin type A have had promising results in a variety of doses and regimens in both traditional and idiopathic FBS [2].

## Conclusions

Even though FBS is a rare medical entity, the very specific pain patterns allow physicians to differ-diagnose it from other causes of gustatory stimuli-evoked pain. Thus, the need to extract a detailed pain history is always present. Patients with FBS and free history of trauma or surgery should be advised to undergo an MRI and CT scan of the head and neck to exclude Eagle syndrome and malignancies arising from the parotid gland, ITF or PPS. The same scan should also be repeated in six to nine months as the mass might not be detectable during the initial examination. The possible involvement of diabetic neuropathy in idiopathic FBS needs to be further investigated.
